# Efficacy and safety of deep brain stimulation for treatment-refractory anorexia nervosa: a systematic review and meta-analysis

**DOI:** 10.1038/s41398-022-02102-w

**Published:** 2022-08-15

**Authors:** Dominika Karaszewska, Patrick Cleintuar, Marloes Oudijn, Anja Lok, Annemarie van Elburg, Damiaan Denys, Roel Mocking

**Affiliations:** 1 Department of Psychiatry, Amsterdam UMC, location Academic Medical Center, University of Amsterdam, Amsterdam, the Netherlands; 2 Faculty of Social Sciences, University of Utrecht, Utrecht, the Netherlands; 3 Rintveld, Center for Eating Disorders, Altrecht Mental Health Institute, Zeist, the Netherlands; 4 Co-eur, Utrecht, the Netherlands

**Keywords:** Neuroscience, Psychiatric disorders

## Abstract

**Background:**

Several pioneering studies investigated deep brain stimulation (DBS) in treatment-refractory anorexia nervosa (AN) patients, but overall effects remain yet unclear. Aim of this study was to obtain estimates of efficacy of DBS in AN-patients using meta-analysis.

**Methods:**

We searched three electronic databases until 1st of November 2021, using terms related to DBS and AN. We included trials that investigated the clinical effects of DBS in AN-patients. We obtained data including psychiatric comorbidities, medication use, DBS target, and study duration. Primary outcome was Body Mass Index (BMI), secondary outcome was quality of life, and the severity of psychiatric symptoms, including eating disorder, obsessive-compulsive, depressive, and anxiety symptoms. We assessed the risk of bias using the ROBINS-I tool.

**Results:**

Four studies were included for meta-analysis, with a total of 56 patients with treatment-refractory AN. Follow-up ranged from 6–24 months. Random effects meta-analysis showed a significant increase in BMI following DBS, with a large effect size (Hedges’s *g* = 1 ∙ 13; 95% CI = 0 ∙ 80 to 1 ∙ 46; *Z*-value = 6 ∙ 75; *P* < 0 ∙ 001), without heterogeneity (*I*^2 ^= 0 ∙ 00, *P* = 0 ∙ 901). Random effects meta-analysis also showed a significant increase in quality of life (Hedges’s *g* = 0 ∙ 86; 95% CI = 0 ∙ 44 to 1 ∙ 28; *Z*-value = 4 ∙ 01, *P* < 0 ∙ 001). Furthermore, DBS decreased the severity of psychiatric symptoms (Hedges’s *g* = 0 ∙ 89; 95% CI = 0 ∙ 57 to 1 ∙ 21; *Z*-value = 5 ∙ 47; *P* < 0 ∙ 001, *I*^2 ^= 4 ∙ 29, *P* = 0 ∙ 371).

**Discussion:**

In this first meta-analysis, DBS showed statistically large beneficial effects on weight restoration, quality of life, and reduction of psychiatric symptoms in patients with treatment-refractory AN. These outcomes call for more extensive naturalistic studies to determine the clinical relevance for functional recovery.

This study is preregistered in PROSPERO,CRD42022295712.

## Introduction

Anorexia nervosa (AN) has an alarming mortality rate [1–3]. About 20% of AN patients remain treatment-refractory to psychotherapy and pharmacological treatment aimed at weight restoration [4]. To ameliorate this grim perspective, there is an urgent need for novel treatment options.

One promising new treatment is deep brain stimulation (DBS). DBS is neuromodulation therapy involving implantation of electrodes at targeted brain areas, which conduct electrical impulses to the brain tissue. These electrical impulses are controlled by a neurostimulator, a device similar to a pacemaker [5]. DBS is already established as an effective treatment for Parkinson’s disease (PD), essential tremor, idiopathic dystonia, epilepsy, and obsessive-compulsive disorder (OCD) [6, 7].

The idea of treating AN with DBS came from serendipitous positive effects that were noted in earlier DBS studies for other indications. In these case reports, patients were being treated with DBS for major depressive disorder (MDD) [8, 9] or OCD [10, 11], while they simultaneously suffered from comorbid AN. Follow-up showed not only a decrease of the MDD or OCD symptoms, but also an improvement of the AN symptoms, including cognitive and emotional symptoms. Patients presented significant improvement in BMI and decreased anxiety and distress in relation to weight gain [8–11].

DBS also holds promise for understanding AN from a pathophysiological perspective. The reward system has been proposed as a key brain circuit in AN [12]. This system regulates motivation and hedonic experience of food intake and provides feedback on the value of a specific food. Several lines of evidence show disturbances in the reward system in AN-patients, including failure to connect appropriate responses to stimuli [13], and limited awareness of intero- and exteroceptive homoeostatic triggers [14, 15]. Moreover, these disturbances in the reward system are linked to formation of habits [16], reflected in repetitive anorectic behaviours and associated altered activation of striatal brain areas [13, 17]. DBS has been shown to directly influence the reward and habit brain circuits, normalizing the aberrant activity associated with psychiatric disorders [18].

Based on these serendipitous clinical observations and pathophysiological insights, pioneering studies tested the effects of DBS in patients with AN. In 2013, Lipsman et al. hypothesized the subcallosal cingulate cortex (SCC) and the nucleus accumbens (NAcc) to be effective DBS targets for AN because they met the following criteria; (1) prominent afferent and efferent connection with the anterior insula, (2) involved in reward-processing, (3) involved in AN-related provocation and imaging studies, and (4) involved in anxiety and dysphoric mood [14]. Indeed, studies observed positive effects of both NAcc and SCC DBS on AN symptoms [6, 9]. Moreover, based on effectiveness in psychiatric disorders phenomenologically related to AN, including treatment-refractory OCD and depression, other targets such as the ventral anterior limb of the internal capsule (vALIC) have been successfully tested in AN [15, 19, 20].

Despite promising trial results, to our knowledge, a meta-analysis on the effects of DBS in AN had yet to be performed. In order to provide an estimate on the overall effect of DBS in AN, we performed such a meta-analysis, combining all available evidence from treatment trials. Based on previous case reports, we hypothesized a beneficial effect of DBS on weight restoration, quality of life, and reduction of psychiatric symptoms in AN patients. Results of this meta-analysis may justify further clinical application in future and more extensive naturalistic research.

## Methods and materials

Following PRISMA guidelines [21], the systematic review and meta-analysis protocol was registered in PROSPERO [22].

### Search strategy

A literature search was performed on MEDLINE, Embase, and PsycInfo, in the period between the last week of August 2020 and up to the 1st of November 2021 (Appendix 1). Publication date was not a restriction.

### Selection process

Two independent reviewers (DMK, PC) screened titles and abstracts of found studies. We included clinical studies investigating the effects of DBS in patients with AN. Duplicates, case reports, and reviews were excluded. Articles were excluded if they did not cover AN and/or DBS, were based on animals, or contained <four participants. We did not exclude studies based on the availability of a control group, studies comparing outcomes before and after DBS will also be included. Inconsistencies were solved by means of discussion, if necessary, with a third and fourth reviewer (MSO, RJTM).

### Critical appraisal and quality assessment

Critical appraisal of all included studies was performed independently in duplicate (DK, PC) by using the ROBINS-I tool (Risk Of Bias In Non-randomised Studies—of Interventions) [23]. Furthermore, we used GRADE [24] for assessing the overall quality of evidence. Inconsistencies were solved by means of discussion.

### Data extraction and outcome data

The extracted data consisted of the following study characteristics: number of participants, number of time points, participants’ characteristics, study duration, and study outcome data. BMI change after DBS was considered our primary outcome. One study used the mean BMI achieved either in the year or in the 3-month prior to surgery, chosen depending on the characteristics of every patient [25]. The combined effect on psychiatric symptoms at the last observation was considered our secondary outcome. In this meta-analysis no other eating disorder related symptoms than BMI could be used as primary outcome data, because of inconsistencies in outcome assessment selection between the included studies. The secondary outcome data was represented using several scales: Yale-Brown-Cornell Eating Disorder Scale [26], Yale-Brown Obsessive-Compulsive Scale (Y-BOCS) [27], Hamilton Anxiety Rating Scale (HAM-A) [28], and Hamilton Depression Rating Scale (HAM-D) [29]. Quality of life was assessed using the Quality of Life Scale [30], SF36 [31], and Eating Disorder Quality of Life [32]. Next to effectiveness data, we also extracted safety data including adverse effects and complications.

### Statistical analyses

#### Main analyses

We used random effect models with Comprehensive Meta-Analysis V3 for our quantitative data synthesis. We used Hedges’ *g* for continuous results, with a 95% confidence interval and two-tailed *p*-values. Sensitivity analyses were performed using pre-operative BMI measures of Villalba et al. instead of the calculated reference BMI value [25]. Forest plots with *I*^*2*^ statistics were used to examine any study heterogeneity.

#### Publication bias

A funnel plot was plotted to assess publication bias. The classic and Orwin’s fail-safe N, Begg and Mazumdar rank correlation, and Egger’s regression intercept were calculated. Should it be deemed necessary Duval and Tweedie’s trim-and-fill method was used to report adjusted values.

## Results

### Study selection

**Fig. 1:**
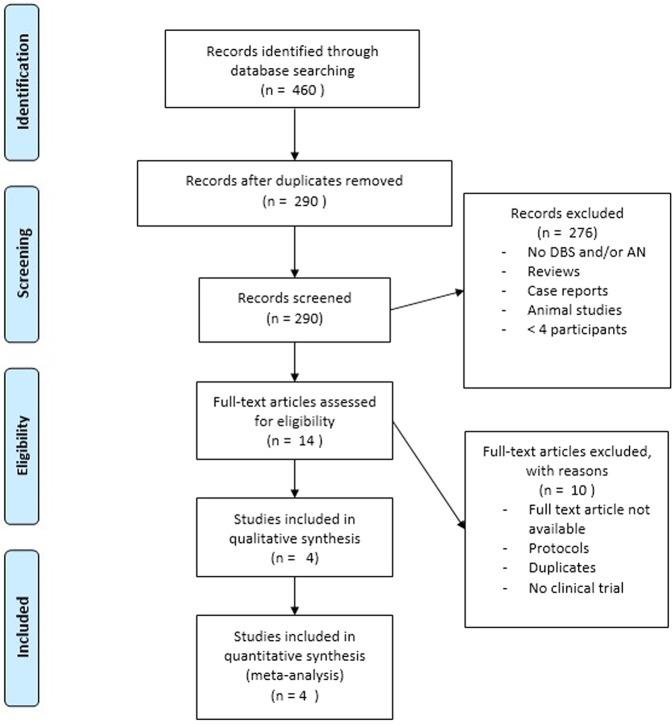
Prisma flow diagram for included studies.

The literature search provided a set of 290 articles. 276 articles were excluded based on title and abstract [Fig. 1]. The full text of the fourteen remaining articles was reviewed. Duplicates and articles that turned out to be protocols were removed from analysis. Four of these fourteen articles were included for meta-analysis. Fig. 1 shows the process of study selection.

### Study characteristics

Four included non-randomized non-controlled clinical trials contained a total of 56 participants. The follow-up period was either 6 [25], 12 [15, 33] or 24 months [4]. The average age of the participants over all studies was 29 ∙ 8 years, with a range of 18–57 years. The mean illness duration over the four included studies was 12 ∙ 8 years. All but one of the participants were female. All patients were diagnosed with AN, with either the restrictive (*n* = 28) or the (binge-)purging subtype (*n* = 28). All participants were defined as treatment-refractory, which for instance included numerous hospital admissions and extensive psychiatric treatment. Most patients suffered from psychiatric comorbidities (*n* = 54). These comorbidities were diagnosed as: MDD (*n* = 33), OCD (*n* = 19), anxiety/generalised anxiety disorder (GAD) (*n* = 11), post-traumatic stress disorder (PTSD) (*n* = 10), panic disorder (*n* = 3), borderline personality disorder (BPD) (*n* = 3), personality disorder not otherwise specified (PD-NOS) (*n* = 2), and substance use disorder (*n* = 2). Supplementary Table 1 shows all study and patients characteristics.

At time of surgery, eight participants were not taking medication, six used one drug and 42 used two drugs or more. The types of drugs consisted of SSRIs (selective and non-selective), benzodiazepine agonists, atypical antipsychotics, antiepileptics, and tetracyclic antidepressants. No major psychopharmacological adjustments were made during the follow-up.

32 out of 56 participants received DBS to the NAcc, 20 patients received DBS to the SCC, and 4 to the vALIC. Three of the studies [4, 15, 33] used either the SCC, NAcc, or the vALIC as the DBS target, while the fourth study [25] used either the SCC or NAcc for their patients, based on their primary comorbidity. 4 out of 8 participants received DBS to the SCC, and 4 to the NAcc in the last mentioned study. Overall, 3 out of 56 participants had their electrodes explanted before the end of follow-up [4, 33, 34].

### Main analyses: effects on BMI

**Fig. 2:**
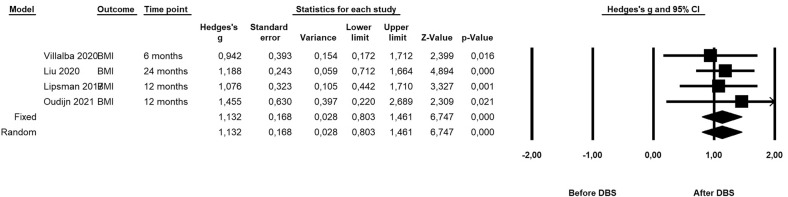
Forrest plot showing meta-analysis effects of DBS on primary outcome BMI.

Random effects meta-analysis showed a significant increasing effect of DBS on primary outcome BMI change after DBS, with a large effect size (Hedges’s *g* = 1 ∙ 13; 95% CI = 0 ∙ 80 to 1 ∙ 46; *Z*-value = 6 ∙ 75; *P* < 0 ∙ 001, Fig. 2), without heterogeneity (*I*^*2*^ = 0 ∙ 00, *P* = 0 ∙ 901).

### Secondary analysis: effects on psychiatric symptom domains

DBS also had a beneficial effect on secondary outcome combined psychiatric symptom severity at last observation (Hedges’s *g* = 0 ∙ 89; 95% CI = 0 ∙ 57 to 1 ∙ 21; *Z*-value = 5 ∙ 47; *P* < 0 ∙ 001, *I*^*2*^ = 4 ∙ 29, *P* = 0 ∙ 371, Supplementary Fig. 2).

#### Eating disorder symptoms

Three non-randomized non-controlled clinical trials [15, 25, 33] assessed eating disorder symptoms. Random effects analyses showed a beneficial main effect of 0 ∙ 98 (Hedges’s *g*; 95% CI = 0 ∙ 28 to 1 ∙ 68; *Z*-value = 2 ∙ 74; *P* = 0 ∙ 006; Supplementary Fig. 3).

#### Symptoms of depression

All studies assessed symptoms related to depression. Random effects analyses showed a beneficial main effect of 0 ∙ 98 (Hedges’s *g*; 95% CI = 0 ∙ 54 to 1 ∙ 41; *Z-*value = 4 ∙ 43; *P* = 0 ∙ 00; Supplementary Fig. 4).

#### Obsessive-compulsive symptoms

All studies assessed obsessive-compulsive symptoms. Random effects analyses showed a beneficial main effect of 0 ∙ 72 (Hedges’s *g*; 95% CI = 0 ∙ 39 to 1 ∙ 06; *Z*-value = 4 ∙ 19; *P* = 0 ∙ 00; Supplementary Fig. 5).

### Symptoms of anxiety

Three non-randomized non-controlled clinical trials [4, 15, 25] assessed symptoms of anxiety. Random effects analyses showed a beneficial main effect of 0 ∙ 85 (Hedges’s *g*; 95% CI = 0 ∙ 38 to 1 ∙ 31; *Z*-value = 3 ∙ 55; *P* = 0 ∙ 00; Supplementary Fig. 6).

### Secondary analysis: quality of life

Three non-randomized non-controlled clinical trials [15, 25, 33] assessed eating disorder symptoms. Random effects analyses showed a beneficial main effect of 0 ∙ 86 (Hedges’s *g*; 95% CI = 0 ∙ 44 to 1 ∙ 28; *Z*-value = 4 ∙ 01; *P* = 0 ∙ 00; Supplementary Fig. 7).

### Adverse events

The most frequently occurring adverse events in the four studies were related to surgery or procedure; pain at incision site within <4 days (*n* = 22), pain at incision site after >4 days (*n* = 5), cutaneous complications (*n* = 4), and to stimulation procedure; hypomanic symptoms (*n* = 3), seizure (*n* = 3), and auto-intoxication (*n* = 3). Supplementary Table 2 lists all reported adverse events, including those possibly but not probably related to the intervention [15]. Other reported adverse events were either defined as unrelated to the intervention and attributed to underlying illness, or had no cause specified. Short-term side-effects like flush and sweating were observed during the programming of the DBS device, but were relieved with adjustment of the parameters.

### Risk of bias and quality of evidence

Risk of bias summary is shown in Supplementary fig. 1. The risk of bias of the objective (BMI) and subjective (psychological outcomes) measurements of the included studies are respectively shown in supplementary Fig. 1a, b. Overall, the risk of bias was moderate for objective outcome measurements and serious for subjective outcome measurements. This was mainly caused by the lack of a control group in the included studies, potentially leading to biases. Furthermore, the studies did not report blinding of both the participants and the researchers, or assessment of the subjective outcomes by an independent party, leading to serious risk of potential bias in measurement of subjective psychological outcomes. No other clear evidence for biases was found.

Supplementary Table 3 shows details of the assessment of quality of the resulting evidence using GRADE. For the more objective outcome BMI, the quality of evidence that deep brain stimulation improves BMI in refractory-treatment AN-patients is considered moderate, whereas for subjective outcomes psychiatric symptoms and quality of life the quality of evidence is deemed low.

### Publication bias

The classic fail-safe *N* was 40, Orwin’s fail-safe *N* was 42 with criterion for a ‘trivial’ standardized difference in means as 0 ∙ 1. This suggested that at least 40 studies without any effect must be reported to decrease the overall effect to a trivial effect. Concerning the Begg and Mazumbar rank correlation test, Kendall’s tau’s with as well as without continuity correction was 0 ∙ 00 (P2-sided = 1 ∙ 00), suggesting no publication bias. Egger’s regression intercept was 0 ∙ 23 (95% confidence interval: −3 ∙ 47 to 3 ∙ 94; P2-sided = 0 ∙ 81), also indicating no publication bias.

**Fig. 3:**
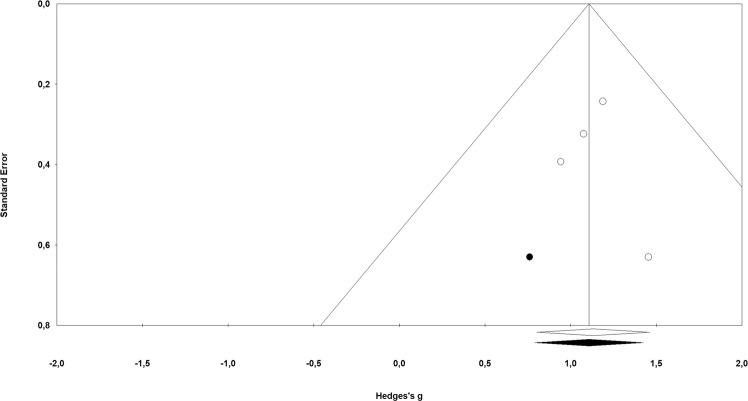
Funnel plot of precision of standard error by Hedges’s g for publication bias.

Duval and Tweedie’s trim-and-fill method used the random-effects model looking for missing studies to the left of the mean, meaning a less favourable effect of deep brain stimulation, showed one study that needed to be trimmed. This resulted in an effect size of 0 ∙ 91 (*Q*-value; 95% CI = 0 ∙ 79 to 1 ∙ 43). Fig. 3 shows the values of the Duval and Tweedie’s trim-and-fill method. Using a fixed effect model, the resulting point estimate did not change.

### Sensitivity analyses

Using the pre-operative BMI instead of the reference BMI for the study of Villalba et al. resulted in a comparable overall effect of DBS on primary outcome BMI change after DBS, with a large effect size (Hedges’s *g* = 0 ∙ 90; 95% CI = 0 ∙ 58 to 1 ∙ 21; *Z*-value = 5 ∙ 60; *P* < 0 ∙ 001).

## Discussion

This first meta-analysis combined all available trials on the effect of DBS in AN. Four non-randomized non-controlled studies provided a total of 56 participants. Random effects meta-analysis showed an improvement in primary outcome BMI, with a large effect size of 1 ∙ 13 and no heterogeneity. Moreover, meta-analysis showed a beneficial effect on overall psychiatric symptom severity, with a comparable large effect size of 0 ∙ 89. All four symptom domains showed a large effect: depressive symptoms (Hedges’s *g* = 0 ∙ 98), obsessive-compulsive symptoms (Hedges’s *g* = 0 ∙ 72), symptoms of anxiety (Hedges’s *g* = 0 ∙ 85), and eating disorder symptoms (Hedges’s *g* = 0 ∙ 98). Furthermore, DBS also improved the quality of life, with a large effect size (Hedges’s *g* = 0 ∙ 86). There was limited evidence for publication bias. The quality of evidence was considered moderate for the more objective primary outcome BMI, and low for the subjective secondary psychological outcome measurements. All in all, results suggest that DBS has statistically large beneficial effects in severe and life-threatening treatment-refractory AN.

Although no statistical heterogeneity was found between included studies, some differences are worth mentioning. It is important to note that the study of Villalba et al. had a relatively short period of follow-up of 6 months. This is particularly noteworthy in comparison to the study of Oudijn et al., where an optimization period was included prior to the maintenance phase leading to a maximal post-operative follow-up of 91 weeks. This shorter follow-up may explain the relatively low post-intervention BMI in Villalba et al., although this did not result in statistical heterogeneity between studies. However, the studies used different targets for DBS, possibly resulting in clinical heterogeneity. Another important difference is that only one study reported psychiatric adverse events related to DBS [15]. Other studies either experienced no psychological adverse events or did not report them systematically.

The risk of bias was considered moderate for objective outcome measurements (BMI) and serious for subjective secondary outcome measurements. This was caused by the lack of a control group, which ethically speaking would be difficult to implement in such a treatment-refractory patient group. A placebo effect could therefore not be excluded. Independent outcome assessors blinded for treatment status could theoretically ameliorate this bias. Nevertheless, we did not find any clear evidence for other biases.

The present meta-analysis showed statistically large effects of DBS in treatment-refractory AN for weight gain, whereas effect sizes otherwise found in literature for pharmacological and psychological treatment seem to be respectively moderate [35, 36] and low [37]. The effects of DBS in AN are in line with effects of DBS in other psychiatric disorders including depression and OCD [38].

The beneficial clinical effects of DBS in this meta-analyses may also improve insight in the pathophysiology of AN. It is hypothesised that DBS creates a reversible lesion to the stimulated area [6, 39]. However, recent evidence suggests that DBS modulates widespread brain network activity, e.g. normalizing neuronal firing in reward circuitry. Due to the uncertainties in the pathophysiology of AN and the working mechanisms of DBS, included trials used diverse stimulation sites, including the NAcc, SCC, and vALIC. However, the effects of the studies were comparable with low heterogeneity. This shows that DBS may be effective at diverse targets, potentially suggesting diverse inroads to normalize aberrant activity in comparable brain circuits. This is in line with the concept of connectomic DBS, where different implementation targets all relate to similar pathophysiologically relevant white matter tracts [40]. Moreover, these effects may be shared with other psychiatric disorders, where similar targets resulted in transdiagnostic beneficial effects [34, 41].

Literature on the biological effects of DBS in AN is sparse. Zhang et al. used FDG-PET to image glucose metabolism in the brain after DBS to the NAcc to identify which brain regions would be affected by DBS in AN [42], and noted a reduction of (hyper)metabolism in the lentiform nucleus, hippocampus, and frontal lobe. Further evidence comes from research other disorders. DBS to the NAcc in treatment-resistant depression showed metabolic decreases in prefrontal subregions, subgenual cingulate region, posterior cingulate cortex, thalamus and caudate nucleus. DBS of the anterior limb of the internal capsule in patients suffering from OCD, resulted in long-term changes in metabolic activity [43]. A decrease of frontal metabolism is a fundamental component of NAcc-DBS mechanism [44]. Moreover, Fridgeirsson et al. found an association in OCD between improvement in mood and anxiety with decreased functional connectivity between the amygdala and insula due to DBS [45]. These circuitries are also involved in AN, presumably, though the precise mechanisms of action of DBS remains to be determined.

### Limitations and strengths

The main limitation of this study was the inability to rule out the presence of a placebo effect. The nature of DBS and particularly AN make it difficult to apply a double-blind study design. Nevertheless, effects, including on the objective outcome BMI, in this severe and extremely treatment-refractory population maintained over a study follow-up of up to 24 months, which argues for consistency and durability. Another limitation is the variation in stimulation targets between the studies. Nevertheless, heterogeneity was low, suggesting that the different stimulation sites have comparable effects. Furthermore, the overall risk of bias of the included studies was moderate to serious. This could be improved by adding an independent assessor to the study. Also the quality of evidence was considered moderate for the more objective primary outcome, and low for the subjective outcome measurements. The pooled results showed statistically large effects suggesting clinical relevance, however more parameters should be taken into account to allow the conclusion of relevant clinical functional improvement. It is important to note that our meta-analysis showed a large beneficial effect of DBS on BMI, however not all patients reached BMI in a normal range at the end of the follow-up. Also, three included studies reported only somatic adverse events related to surgery or stimulation. Only one study reported psychiatric adverse events related to DBS [15]. Finally, no international consensus has been reached on the definition of response to treatment or remission in anorexia nervosa in general, and of response to DBS in AN in particular. The included studies used heterogeneous definitions of response to DBS, therefore responder rates could not be determined. This study again emphasizes the need for such a consensus, as this would allow clinicians to assess the efficacy of this procedure.

A major strength of this study was that it is the first meta-analysis of the effect of DBS in treatment-refractory AN. Thereby, we provide an overall estimate of the size of the effect and the strength and quality of the evidence for the efficacy of DBS in treatment-refractory AN.

### Research implications

Results from this meta-analyse provide several new inroads for future research. A first point of focus are the differences and similarities of the diverse DBS targets that have been applied. Studies might focus on clinical and biological differences in effects, e.g. by applying advanced clinical phenotyping and diverse biological assessments including advanced (connectomic) neuroimaging.

A second issue is prediction of response to improve patient selection. It would be helpful to identify factors that may predict response to DBS treatment in AN, and test whether the predictive value is strong enough for clinical implementation [46].

A third aspect is the combination with psychotherapy. DBS studies in psychiatry for other indications suggested that DBS may improve the response to psychotherapeutic interventions. It would be worthwhile to test whether this is also the case for AN-patients, and whether psychotherapy may increase the effectiveness of DBS.

An international database, including up-to-date naturalistic data from all AN-patients that are treated with DBS worldwide, may substantially increase power to test for subgroup effects.

### Clinical implications

It is striking to note that deep brain stimulation, being effective in several psychiatric disorders, is relatively understudied in anorexia nervosa, being the world’s most lethal psychiatric disorder. However, it needs to be noted that, as of yet, both the mechanism of action of DBS and the pathophysiology of AN are not fully understood. To be able to understand and treat AN, more insight is needed in the complex dynamics in which this psychiatric disorder comes to expression. Using DBS targeting different brain areas gets us closer to this understanding or even finding the root of this complex disorder.

Of note, other forms of neuromodulation (i.e. electro-convulsive therapy) also show promising results in anorexia nervosa, particularly on symptoms of depression [47].

Our results suggest that DBS can be an effective last-resort treatment option in severe treatment-refractory AN. Despite the invasive nature of the procedure and the risk of side-effects, we suggest that there is a clinical indication for DBS in selected cases of AN, under strict monitoring and scientific evaluation of effects. Target selection should be based on the available experience of the involved neurosurgeon and psychiatric treatment team. After DBS, the potential effect of concomitant psychological and/or pharmacological therapy should be re-evaluated.

## Conclusion

This meta-analysis demonstrated a statistically large beneficial effect of DBS on weight restoration, quality of life, and psychiatric symptoms severity in patients with treatment-refractory AN. Adverse effects were related to surgery and stimulation. The size of the beneficial effects suggest potential clinical relevance as a last-resort treatment option in severe and life-threatening AN. Future, more extensive, naturalistic research could strengthen conclusions regarding clinical relevance and incorporation in guidelines, also in relation to other forms of neuromodulation. These promising outcomes form new inspiration for future research, and may provide a more hopeful perspective for patients that did not yet respond sufficiently to other forms of therapy.

### Registration and protocol

The systematic review and meta-analysis protocol was preregistered in PROSPERO under study’s registration number: CRD42022295712.

## Supplementary information

Supplementary Information
