# Supplementary material for: Efficacy and safety of deep brain stimulation for treatment-refractory anorexia nervosa: a systematic review and meta-analysis
Source: Transl Psychiatry. 2022 Aug 15;12:333. doi: 10.1038/s41398-022-02102-w (PMC9378729; doi:10.1038/s41398-022-02102-w)
Supplement: Supplementary file 1 — Supplementary Information [file 41398_2022_2102_MOESM1_ESM.docx]

**Supplementary Information**

**Supplementary Table 1. Study characteristics of included studies**

|  | Villalba et al. (N=8) | Liu et al. (N=28) | Lipsman et al. (N=16) | Oudijn et al. (N=4) | **Total / Mean**  **(N=56)** |
| --- | --- | --- | --- | --- | --- |
| **Mean age in years (range)** | 41 (SD = 16) | 23 (SD = 4) | 34 (SD = 8) | 39 (SD = 10) | **30 (18-57)** |
| **Follow-up period** | 6 months | 24 months | 12 months | 12 months* |  |
| **Mean illness duration in years** | 25 (SD = 11) | 5 (3 – 10) | 18 (SD = 6) | 21 (SD = 3) | **13 (SD = 9)** |
| **Percentage female patients** | 87∙5% | 100% | 100% | 100% | **98∙2%** |
|  |  |  |  |  |  |
| **Restrictive subtype** | 6 | 13 | 9 | 0 | **28 (50%)** |
| **(Binge-)purging subtype** | 2 | 15 | 7 | 4 | **28 (50%)** |
|  |  |  |  |  |  |
| **Suffering from psychiatric comorbidities (percentage)** | 8 (100%) | 28 (100%) | 14 (87∙5%) | 4 (100%) | **54 (96∙4%)** |
| **MDD** | 7 (87∙5%) | 12 (42∙9%) | 12 (75%) | 2 (50%) | **33 (58∙9%)** |
| **OCD** | 3 (37∙5%) | 9 (32∙1%) | 6 (37∙5%) | 1 (25%) | **19 (33∙9%)** |
| **GAD / severe anxiety** | 0 | 7 (25%) | 3 (18∙8%) | 1 (25%) | **11 (19∙6%)** |
| **PTSD** | 0 | 0 | 10 (62∙5%) | 0 | **10 (17∙9%)** |
| **Panic disorder** | 3 (37∙5%) | 0 | 0 | 0 | **3 (5∙4%)** |
| **BPD** | 0 | 0 | 2 (12∙5%) | 1 (25%) | **3 (5∙4%)** |
| **PD-NOS** | 0 | 0 | 0 | 3 (75%) | **3 (5∙4%)** |
| **SUD** | 0 | 0 | 1 (6∙3%) | 1 (25%) | **2 (3∙6%)** |
|  |  |  |  |  |  |
| **No medication use** | 1 (12∙5%) | 0 | 7 (43∙8%) | 0 | **8 (14∙3%)** |
| **1 drug** | 4 (50%) | 1 (3∙6%) | 1 (6∙3%) | 0 | **6 (10∙7%)** |
| **2 or 2> drugs** | 3 (37∙5%) | 27 (96∙4%) | 8 (50%) | 4 (100%) | **42 (75%)** |
|  |  |  |  |  |  |
| **DBS Target** | NAcc (n=4) or SCC (n=4) | NAcc | SCC | vALIC | **-** |
|  |  |  |  |  |  |
| **BMI (kg/m3); average baseline** | 12∙67 (SD = 1∙64) | 13∙01 (SD = 1∙86) | 13∙83 (SD = 1∙49) | 12∙44 (SD = 0∙52) | **12∙99 (0∙61)** |
| **BMI (kg.m3); post intervention**** | 13∙98 (SD = 2∙05) | 17∙73 (SD = 3∙54) | 17∙34 (SD = 3∙40) | 17∙76 (SD = 2∙27) | **16∙70 (1∙83)** |
|  |  |  |  |  |  |
| **Y-BOCS; at baseline** | 16∙50 (SD = 7∙69) | 20∙46 (SD = 8∙86) | 27∙88 (SD = 8∙57) | - | **21∙61 (5∙78)** |
| **Y-BOCS; post intervention **** | 12∙75(SD = 11∙76) | 13∙04 (SD = 9∙61) | 19∙71 (SD = 10∙31) | - | **15∙17 (3∙94)** |
| **HAM-A; at baseline** | 13∙63 (SD = 6∙30) | 21∙39 (SD = 8∙54) | - | 29∙25 (SD = 2∙46) | **21∙42 (7∙81)** |
| **HAM-A; post intervention**** | 10∙94 (SD = 11∙84) | 12∙63 (SD = 9∙18) | - | 15∙25 (SD = 1∙11) | **12∙94 (2∙17)** |
| **HAM-D; at baseline** | 15∙38 (SD = 5∙52) | 26∙93 (SD = 11∙97) | 19∙40 (SD = 6∙76) | 27∙25 (SD = 2∙02) | **22∙24 (5∙84)** |
| **HAM-D; post intervention**** | 10∙50 (SD = 9∙87) | 15∙93 (SD = 12∙33) | 8∙79 (SD = 7∙64) | 17∙25 (SD = 1∙67) | **13∙12 (4∙11)** |
| **Analyses** | PP | PP | PP | PP | **-** |

**Abbreviations:** N: number of subjects; AN: Anorexia Nervosa; DBS: Deep brain stimulation; MDD: Major depressive disorder; OCD: Obsessive-compulsive disorder; GAD: Generalized anxiety disorder; PTSD: Post-traumatic stress disorder; BPD: Borderline personality disorder; PD-NOS: Personality disorder not otherwise specified; SUD: Substance use disorder; NAcc: Nucleus accumbens; SCC: Subcallosal cingulate cortex; vALIC: Ventral anterior limb of the internal capsule; YBOCS: Yale-Brown Obsessive-Compulsive Scale; HAM-A: Hamilton Anxiety Rating Scale; HAM-D: Hamilton Depression Rating Scale; PP: per protocol. * After surgery DBS parameters were optimized for 3 weeks with DBS-off and a minimum of 12 weeks with DBS-ON. After optimization phase, the 12 months long maintenance phase followed after. ** The elapsed time for the variables measured after the intervention (BMI, Y-BOCS, HAM-A, HAM-D) is the same as the follow-up period.

**Supplementary Table 2. Number of patients experiencing adverse events probably or possibly related to DBS as reported in the studies**

|  | **Villalba et al (N=8)** | **Liu et al (N=28)** | **Lipsman et al (N=16)** | **Oudijn et al (N=4)** | **Total / Mean**  **(N=56)** |
| --- | --- | --- | --- | --- | --- |
| No adverse events | 5 (62∙5%) | 6 (21∙4%) | 5 (31∙3%) | 0 (0%) | **16 (28∙6%)** |
| Cutaneous complications | 3 (5∙4%) | - | 1 (1∙8%) | - | **4 (7∙1%)** |
| Pain at incision site (<4d) | - | 22 (39∙3%) | - | - | **22 (39∙3%)** |
| Pain at incision site (>4d) | - | - | 5 (8∙9%) | - | **5 (8∙9%)** |
| Intra-operative panic attack | - | - | 1 (1∙8%) | - | **1 (1∙8%)** |
| Hypomanic/manic symptoms | - | - | - | 3 (5∙4%) | **3 (5∙4%)** |
| Hypophosphatemia | - | - | 1 (1∙8%) | - | **1 (1∙8%)** |
| Nausea | - | - | 1 (1∙8%) | - | **1 (1∙8%)** |
| QT prolongation | - | - | 1 (1∙8%) | - | **1 (1∙8%)** |
| Seroquel overdose | - | - | 1 (1∙8%) | - | **1 (1∙8%)** |
| Increased lead impedance | - | - | 1 (1∙8%) | - | **1 (1∙8%)** |
| Worsening mood | - | - | 1 (1∙8%) | - | **1 (1∙8%)** |
| Refeeding delirium | - | - | 1 (1∙8%) | - | **1 (1∙8%)** |
| Pancreatitis | - | - | 1 (1∙8%) | - | **1 (1∙8%)** |
| Seizure | - | - | 2 (3∙6%) | 1*(1∙8%) | **3 (5∙4%)** |
| *Auto-intoxication | - | - | - | 3 (5∙4%) | **3 (5∙4%)** |
| *Self-destructive behavior | - | - | - | 2 (3∙6%) | **2 (3∙6%)** |
| *Aggressive behavior | - | - | - | 1 (1∙8%) | **1 (1∙8%)** |
| *Alcohol consumption | - | - | - | 1 (1∙8%) | **1 (1∙8%)** |
| *Water intoxication | - | - | - | 2 (3∙6%) | **2 (3∙6%)** |
| *Migraine | - | - | - | 1 (1∙8%) | **1 (1∙8%)** |
| *Pain at battery site | - | - | - | 1 (1∙8%) | **1 (1∙8%)** |
| *Severe auto mutilation | - | - | - | 1 (1∙8%) | **1 (1∙8%)** |
| **Hyponatraemia | - | - | 1 (1∙8%) | - | **1 (1∙8%)** |
| **Hypokalaemia | - | - | 1 (1∙8%) | - | **1 (1∙8%)** |

**Abbreviations**: N: number of subjects; d: days; *possibly related, but not probable **Probably due to underlying illness

**Supplementary Table 3. Quality of the evidence of analyses on the effect of DBS on refractory-treatment AN patients**

| **Analysis** | **Study design** | **Risk of bias** | **Inconsistency** | **Indirectness** | **Imprecision** | **Publication bias** | **Effect size** | **Final GRADE score** |
| --- | --- | --- | --- | --- | --- | --- | --- | --- |
| BMI | +3, non-RCTs | -1 | 0, no heterogeneity | 0 | 0 | 0, undetected | +1 | ⊕⊕⊕⊝  moderate |
| Psychiatric symptoms | +3, non-RCTs | -2 | 0, no heterogeneity | 0 | 0 | 0, undetected | +1 | ⊕⊕⊝⊝  low |

**Supplementary figure 1a. Risk of bias of objective measurements of included studies (BMI)**

**
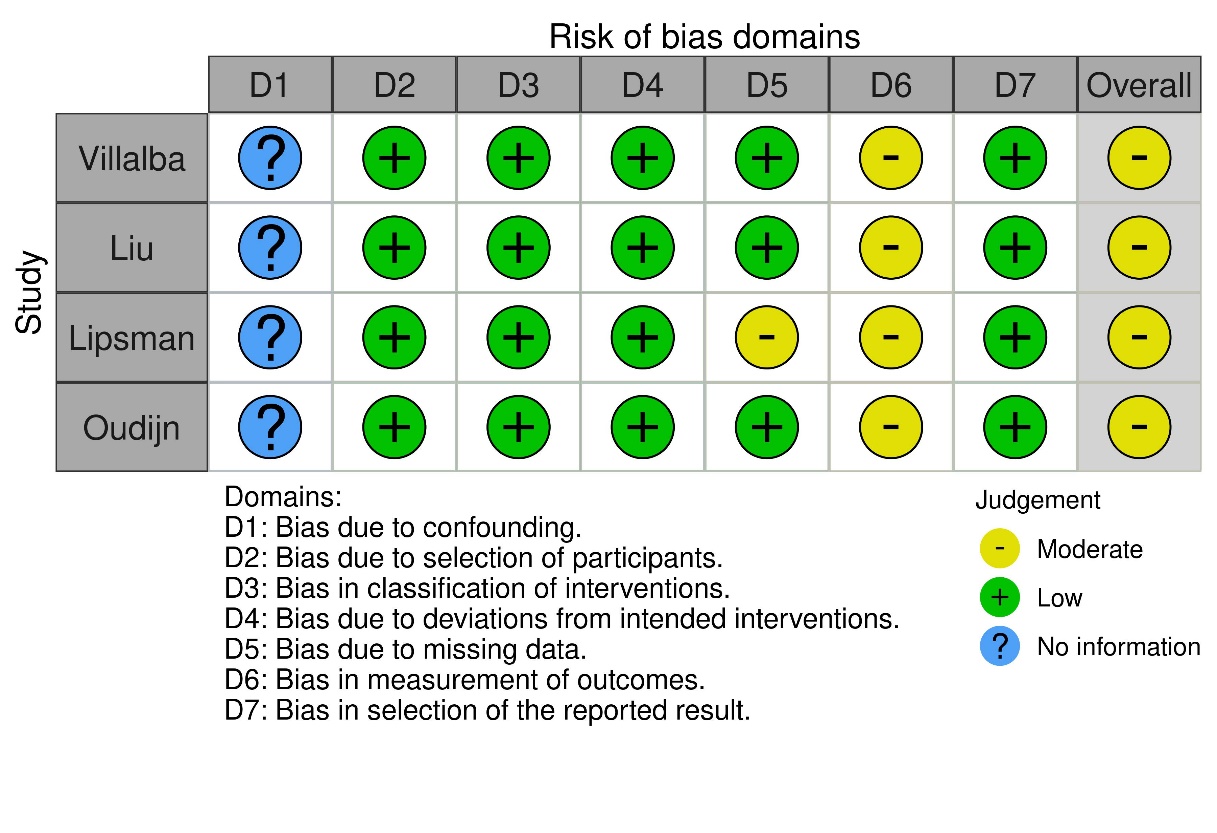
**

**Supplementary figure 1b. Risk of bias of subjective measurements of included studies (psychiatric symptom severity)**

**
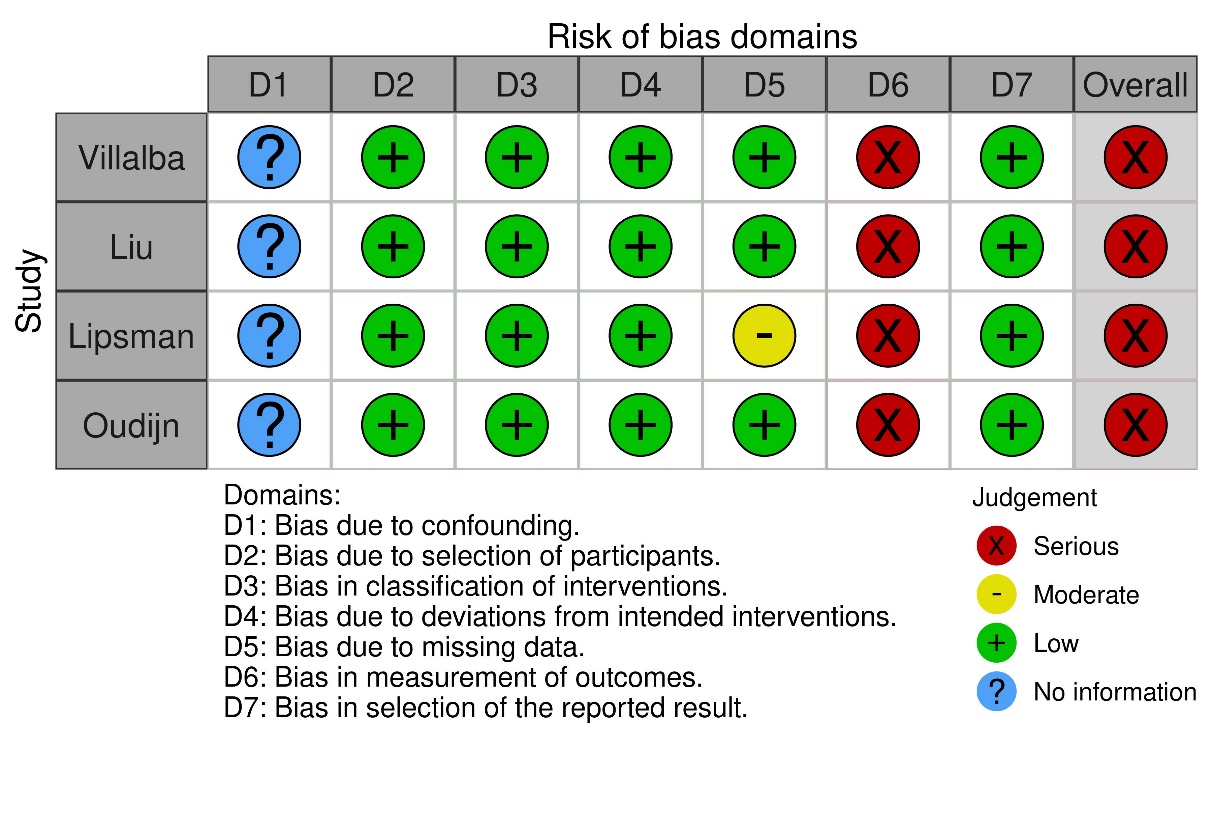
**

**Supplementary Figure 2. Forrest plot showing meta-analysis effects of DBS on secondary outcome combined psychiatric symptom severity**

**
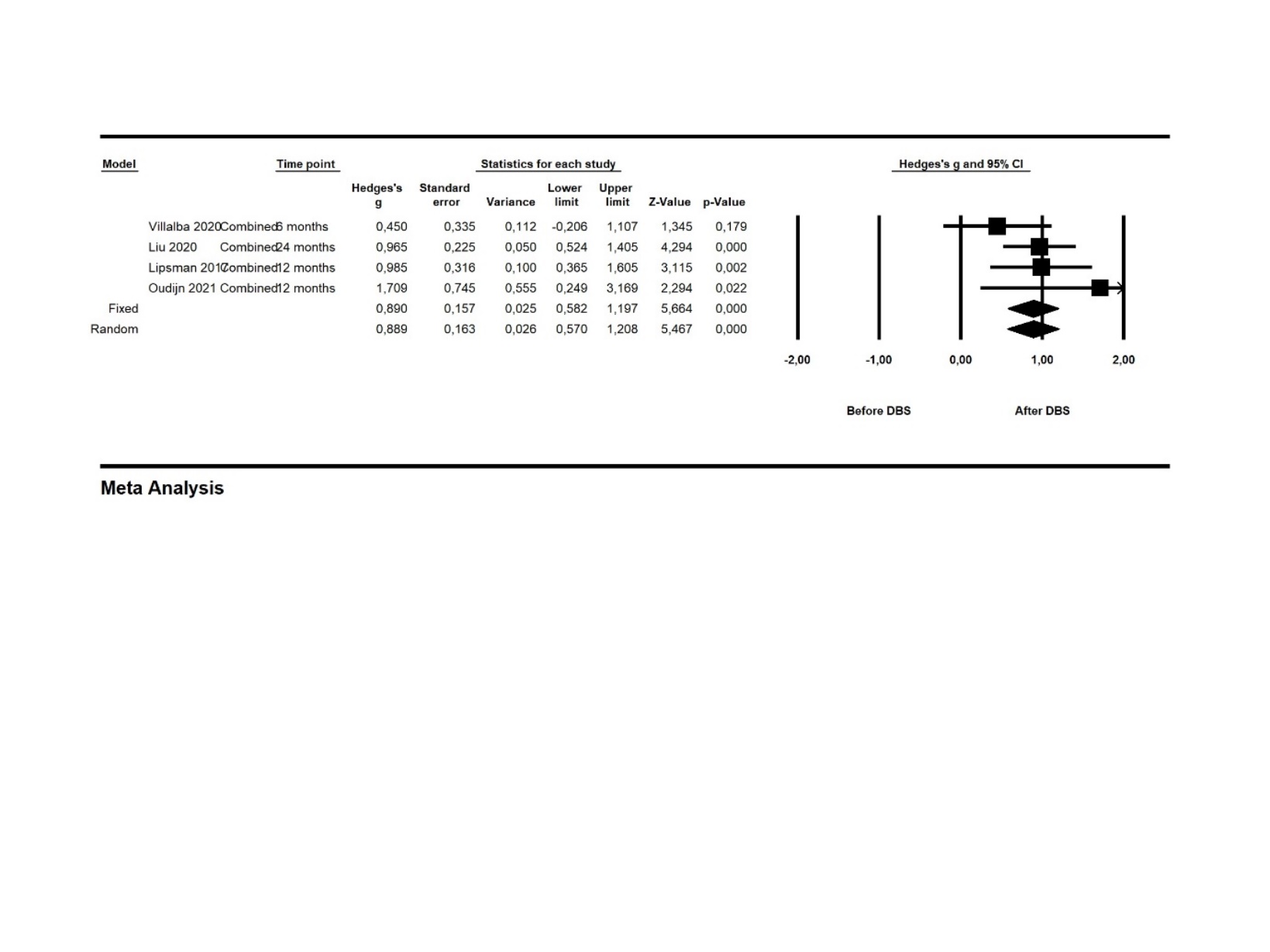
**

**Supplementary Figure 3. Forrest plot showing meta-analysis effects of DBS on eating disorder symptoms**

**
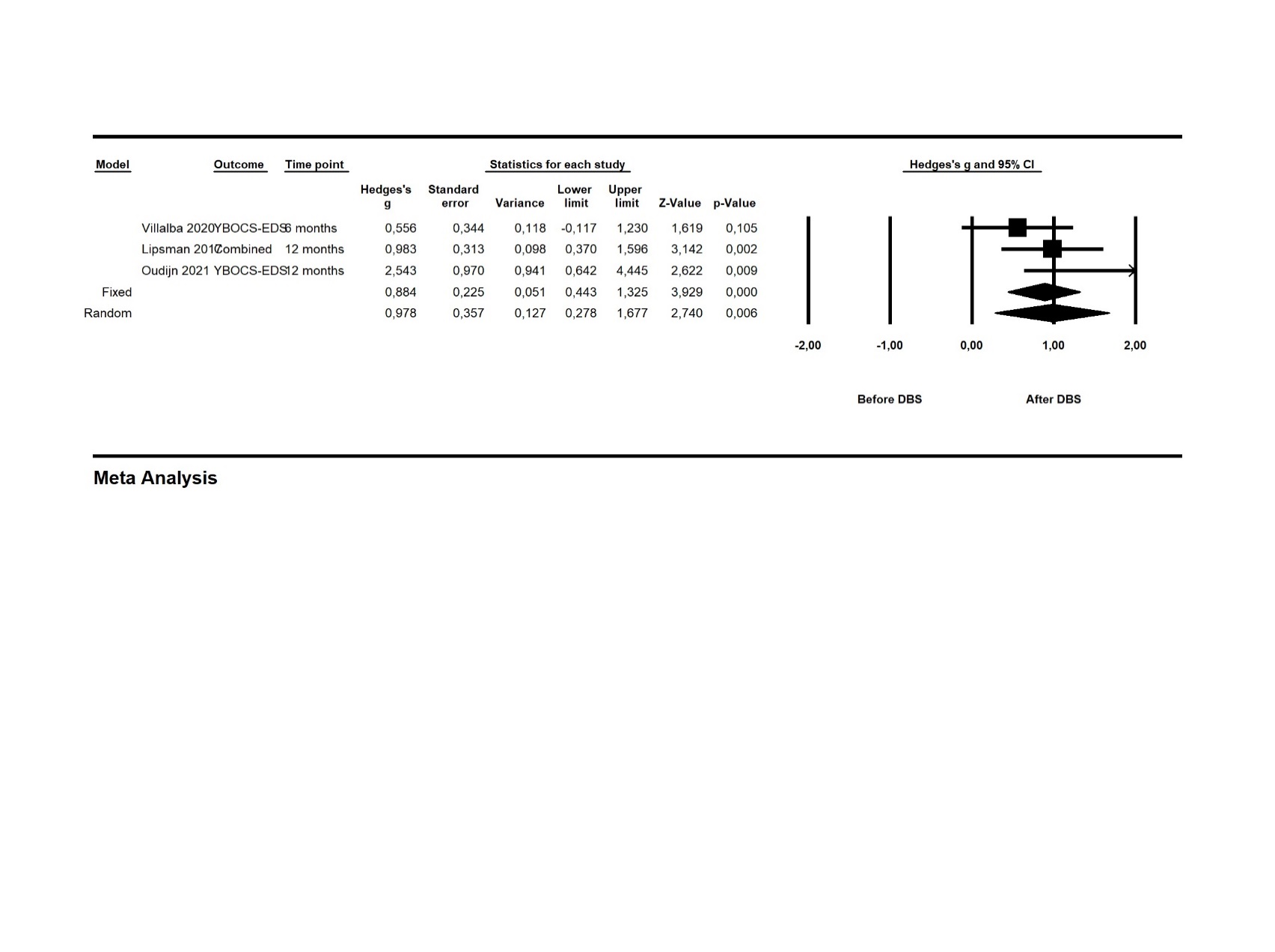
**

**Supplementary Figure 4. Forrest plot showing meta-analysis effects of DBS on symptoms of depression**

**
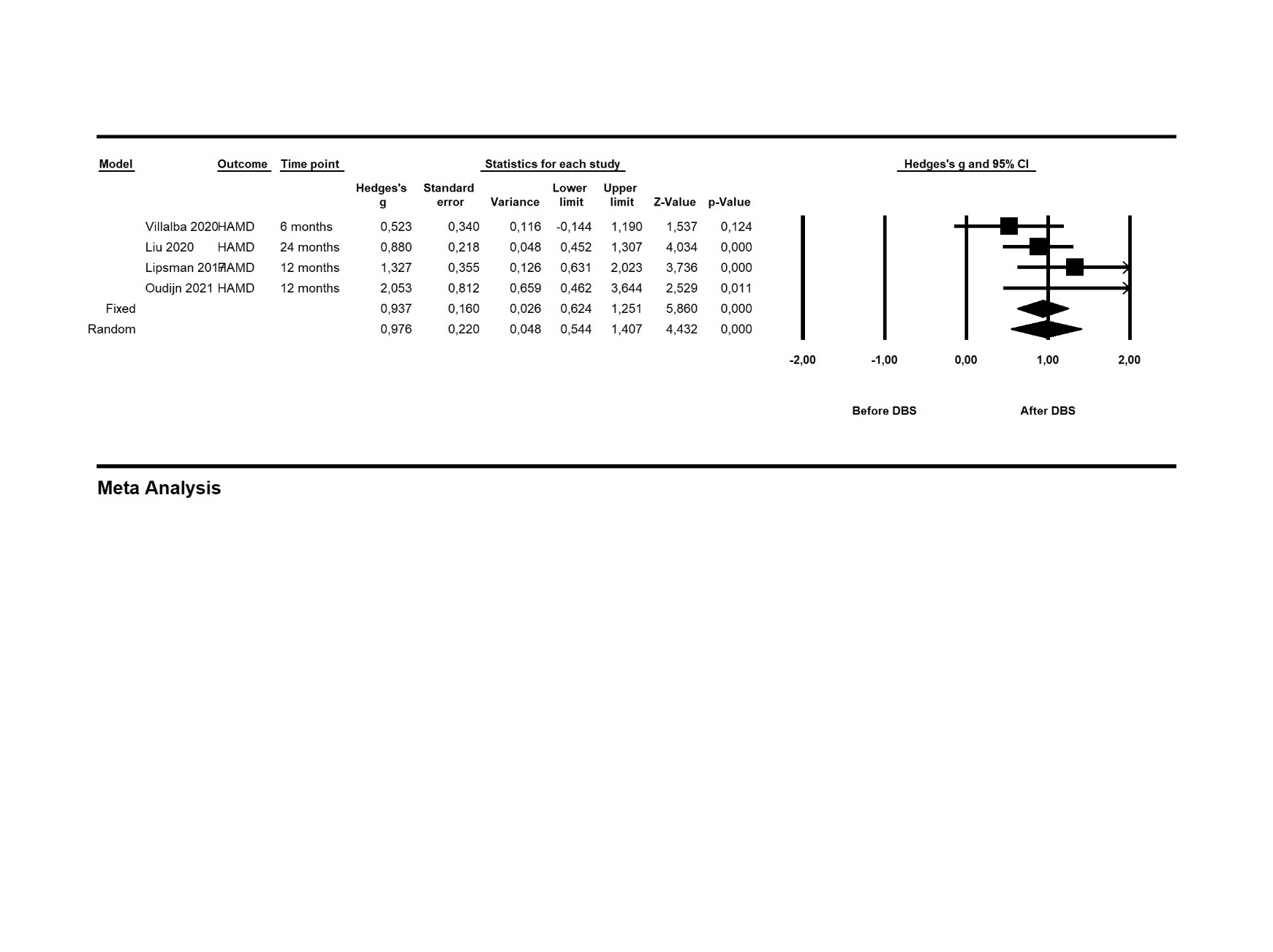
**

**Supplementary Figure 5. Forrest plot showing meta-analysis effects of DBS on obsessive-compulsive symptoms**

**
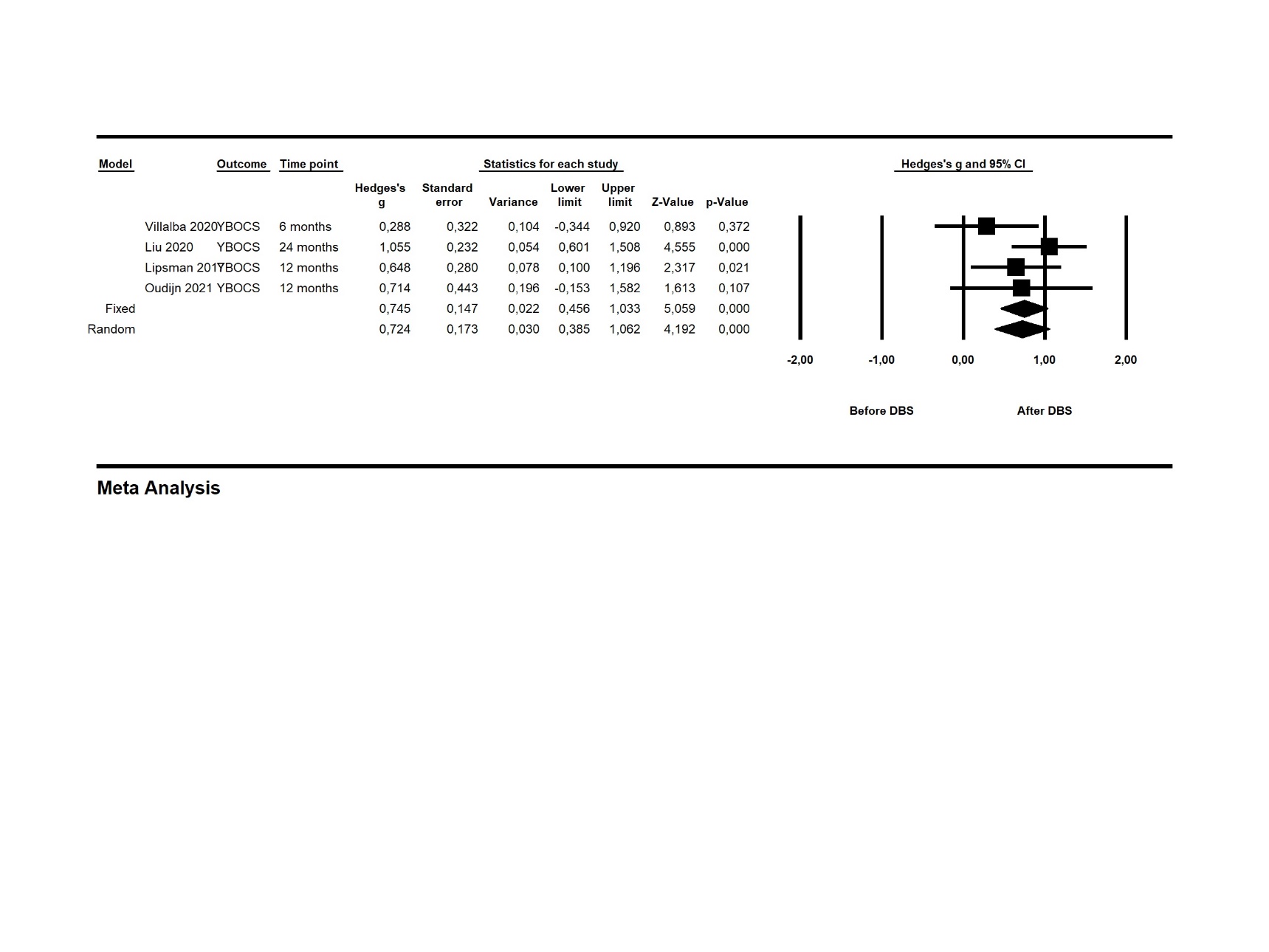
**

**Supplementary Figure 6. Forrest plot showing meta-analysis effects of DBS on symptoms of anxiety**


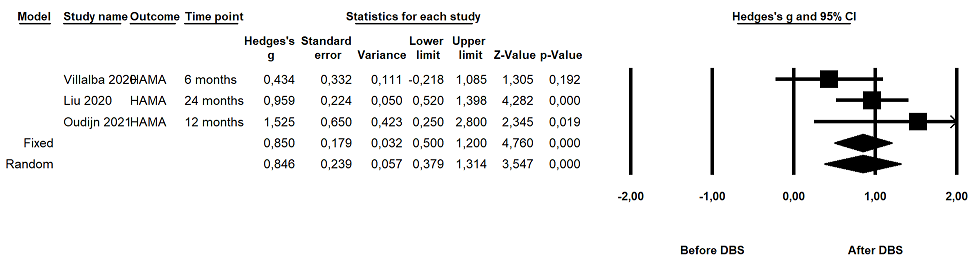


**Supplementary Figure 7. Forrest plot showing meta-analysis effects of DBS on quality of life**

**
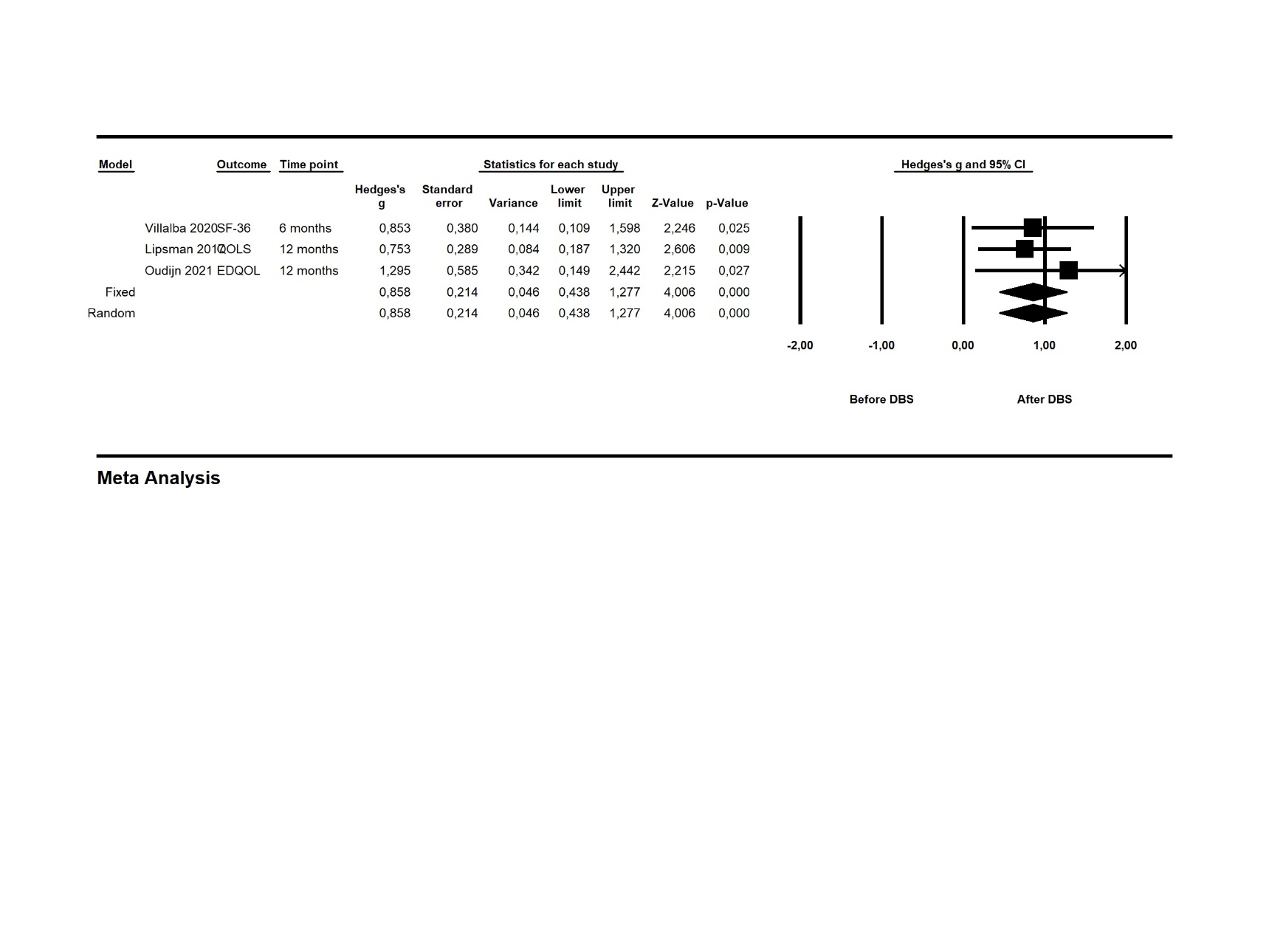
**

**Appendix 1. Search (MEDLINE, Embase, PsycINFO)**

1 exp anorexia nervosa/ 47913

2 anorexi*.ti,ab,id. 105216

3 exp deep brain stimulation/ 59871

4 DBS.ab,ti. 35426

5 (stimul* adj3 (brain or deep)).ab,ti. 72708

6 1 or 2 113152

7 3 or 4 or 5 107874

8 6 and 7 460

9 remove duplicates from 8 290

**Appendix 2. List of studies excluded at full-text screening stage**

| **Study** | **Reason of exclusion** |
| --- | --- |
| A randomized trial of deep brain stimulation to the subcallosal cingulate and nucleus accumbens in patients with treatment-refractory, chronic, and severe anorexia nervosa: Initial results at 6 months of follow up.  Martinez G.V., Justicia A., Salgado P., Gines J.M., Guardiola R., Cedron C., Polo M., Delgado-Martinez I., Medrano S., Manero R.M., Conesa G., Faus G., Grau A., Elices M., Perez V. | Same study as Villalba et al. |
| Nucleus accumbens deep brain stimulation for treatment-refractory anorexia nervosa: A two-year follow-up study.  Hu K., Sun B. | Conference abstract, same study as Liu et al. |
| Study protocol: Using deep-brain stimulation, multimodal neuroimaging and neuroethics to understand and treat severe enduring Anorexia Nervosa.  Park R.J., Scaife J.C., Aziz T.Z. | Study protocol |
| Subcallosal cingulate deep brain stimulation for treatment-refractory anorexia nervosa: A phase 1 pilot trial.  Lipsman N., Woodside D.B., Giacobbe P., Hamani C., Carter J.C., Norwood S.J., Sutandar K., Staab R., Elias G., Lyman C.H., Smith G.S., Lozano A.M. | Phase I of included study in meta-analysis, duplicate |
| Metabolic imaging of deep brain stimulation in anorexia nervosa: A 18F-FDG PET/CT study.  Zhang H.-W., Li D.-Y., Zhao J., Guan Y.-H., Sun B.-M., Zuo C.-T. | Metabolic imaging as outcome measure |
| Six women pilot deep brain stimulation for intractable anorexia nervosa.  Anonymous | Anonymous authors, note not an article, same patients as in Lipsman et al. |
| Deep brain stimulation of the subcallosal cingulate area for treatment-refractory anorexia nervosa: Phase I pilot trial.  Lipsman N., Woodside B., Giacobbe P., Hamani C., Lozano A.M. | Phase I of included study in meta-analysis, duplicate |
| Metabolic imaging of deep brain stimulation in anorexia nervosa: An 18F-FDG PET/CT study.  Zhang H. | Metabolic imaging as outcome measure, duplicate |
| Phase I trial of deep brain stimulation of the subcallosal cingulum for treatment-refractory anorexia nervosa.  Lipsman N., Woodside B., Giacobbe P., Hamani C., Lozano A.M. | Duplicate, phase I |
| Deep brain stimulation for anorexia nervosa - authors' reply.  Lipsman N; Woodside DB; Lozano AM. | Authors reply, comment |
